# The complete chloroplast genome and phylogenetic analysis of *Persicaria jucunda* (Meisn.) Migo (Polygonaceae)

**DOI:** 10.1080/23802359.2025.2457450

**Published:** 2025-01-28

**Authors:** Peixuan Xu, Jinxi Yao, Chunmin Mao, Junrong Zhu, Yao Zhao

**Affiliations:** ^a^ Innovation Group of Orchid Conservation and Utilization, Yunnan Forestry Technological College, Kunming, China; ^b^ Yunnan Key Laboratory of Plateau Wetland Conservation, Restoration and Ecological Services, Southwest Forestry University, Kunming, China; ^c^ Dianchi Lake Ecosystem Observation and Research Station of Yunnan Province, Southwest Forestry University, Kunming, China; ^d^ School of Ecology and Environmental Science, Yunnan University, Kunming, China

**Keywords:** *Persicaria jucunda*, chloroplast genome, Polygonaceae, phylogenetic analysis

## Abstract

*Persicaria jucunda* is a plant distributed at meadow or wetland. Our study reports the complete chloroplast genome. The chloroplast genome of *P. jucunda* is a typical tetrameric structure with a total length of 159,843 bp, containing a large single-copy (LSC) region of 84,350 bp, a small single-copy (SSC) region of 13,151 bp, and two inverted repeat regions (IRs) of 31,171 bp. The total GC content was 38.2%, 36.5% for the LSC region, 33.2% for the SSC region, and 41.5% for the IR region. The *P. jucunda* chloroplast genome contains 128 genes, including 83 protein-coding genes, 37 tRNA genes, and eight rRNA genes. Phylogenetic analysis based on 36 chloroplast genomes showed similarity to *P. foliosa* and *P. kawagoeana* among the species analyzed. The chloroplast genome provides a valuable genetic resource for phylogenetic studies.

## Introduction

*Persicaria jucunda* (Meisn.) Migo (1939) is a member of the genus *Persicaria* in the family Polygonaceae (Miyamoto et al. 2004). *P. jucunda* is an annual herb that occurs in grassy slopes, moist valleys, and along ditches at sea level to 2000 m in Sichuan, Yunnan, Zhejiang, China. *P. jucunda* reaches up to 60–90 cm tall. Stems erect, usually prostrate at base, glabrous, much branched. Petiole 3–6 mm; leaf blade elliptic-lanceolate, both surfaces sparsely appressed-hispidulous or subglabrous, shortly ciliate at margin, base cuneate, margin entire, apex acuminate; ocrea brownish, tubular, 5–10 mm, membranous, sparsely appressed hispidulous, apex truncate, cilia 6–11 mm. Inflorescence terminal or axillary, spicate, dense, 3–6 cm; bracts green, funnel-shaped, cilia 1.5–2 mm, each bract 3–5-flowered. Pedicels longer than bracts, 7–8 mm. Perianth pinkish or white, 5-parted; tepals oblong, 2–3 mm. Stamens 7 or 8, shorter than perianth. Styles 3, connate to below middle, longer than perianth, or stamens longer than perianth and styles shorter than perianth on separate plant; stigmas capitate (Miyamoto et al. 2004). The classification of *P. jucunda* is currently highly controversial. In the classification of the genus *Polygonum*, some scholars have divided it into 11 independent genera. However, due to the lack of clear distinctions among these groups, other researchers advocate for retaining it as a single genus. In China, apart from recognizing *Fagopyrum*, *Antenoron*, *Reynoutria*, and *Fallopia* as independent genera, the remaining groups are still merged into a single genus. These complex taxonomic issues and the history of classification correspond to the diverse and intricate life histories and evolutionary trajectories of the plants (Meissner 1857; Gross 1913; Ronse and Akeroyd 1988). The phylogenetic position of *P. jucunda* can be determined from the chloroplast genome sequence. No chloroplast genome sequence analysis of *P. jucunda* has been reported. In this study, we report the complete chloroplast genome sequence of *P. jucunda* and elucidate its phylogenetic relationships.

## Materials and methods

**Figure 1.**
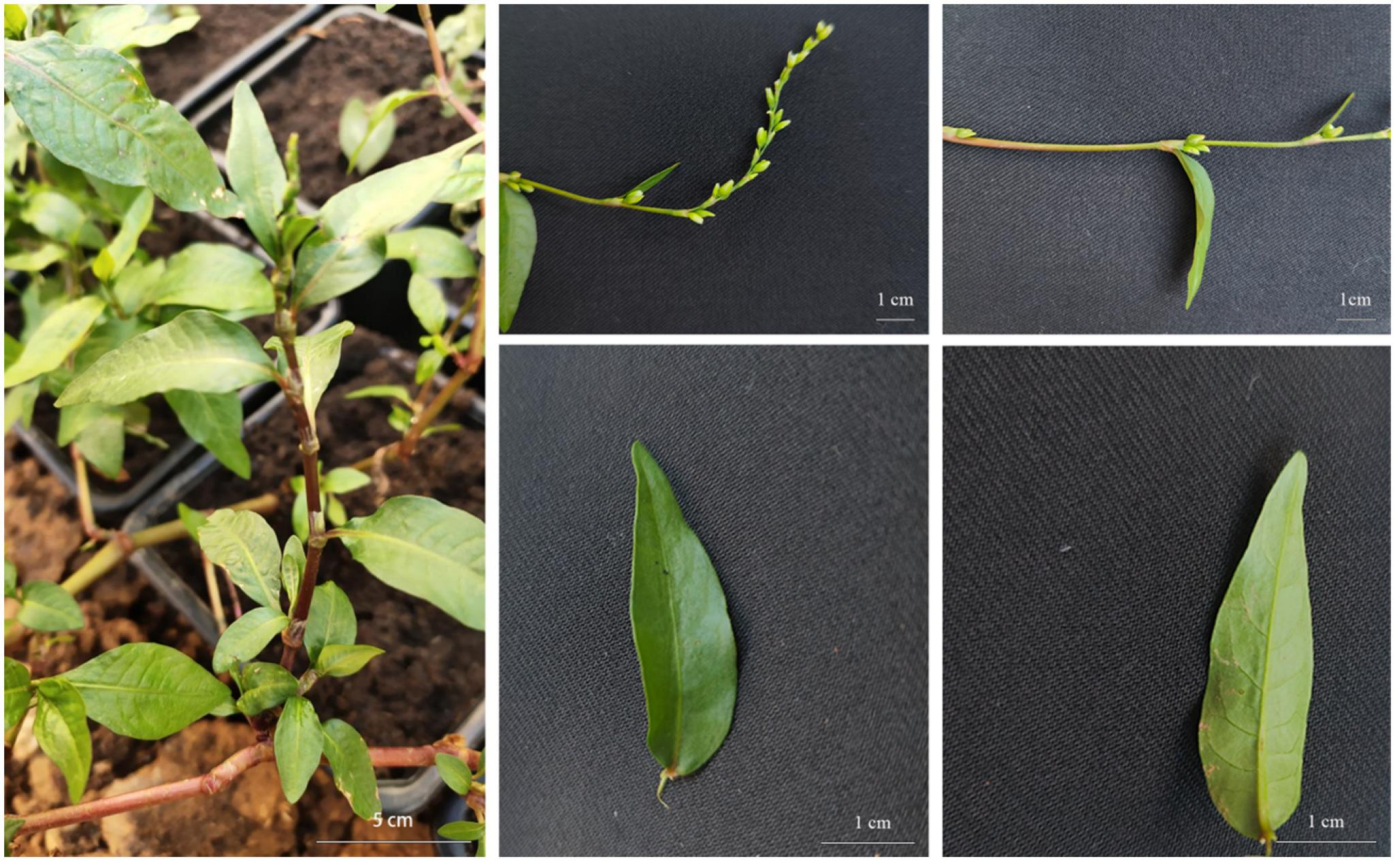
The morphological characteristics of *P. jucunda*. Overall morphological characteristics of the plant, inflorescence traits, leaf characteristics, and perianth segment traits. Photos taken by Yao Zhao in common-garden in Chengjiang, Yunnan Province, China (24°37′40.92″N, 102°51′16.53″E, altitude 1416 m).

Voucher specimens of *P. jucunda* were collected from Yunnan Province (24°37′40.92″N, 102°51′16.53″E) and deposited in the School of Wetland of Southwest Forestry University (HSFU, Zhaoyao-20210004); contact: Zhao Yao, e-mail: zhaoyao1996xs@126.com (Figure 1). Total DNA was extracted from *P. jucunda* leaves, and the sequencing library was sequenced using the Illumina NovaSeq 6000 platform. The chloroplast genome was assembled de novo using the software GetOrganelle V 1.7.7.0 (Jin et al. 2020). The chloroplast genome of the plant *B. vivipara* was used as a reference sequence (GenBank accession number: NC_060351) and annotated using the software PGA (Qu et al. 2019). And the chloroplast genome was manually checked for correction using the software Geneious R9. A circular chloroplast genome atlas with default settings was constructed using the program OGDRAW v1.3.1 (Greiner et al. 2019). In the DnaSP software, nucleotide diversity was calculated using sliding windows with a window length of 400 bp and a step size of 200 bp to evaluate the divergence of the five *Persicaria* cp genome sequences.

Several barrels of developmental analyses were performed using the chloroplast genome sequence of *P. jucunda* and the chloroplast genomes of 33 Polygonaceae plants downloaded from GenBank, with the chloroplast genomes of two Plumbaginaceae as outgroups. Sequence alignment of 36 genomes was performed using MAFFT (Katoh and Standley 2013). The optimal model was selected using ModelFinder (Kalyaanamoorthy et al. 2017), and The IQ-TREE 1.6.12 (Nguyen et al. 2015) was used for maximum-likelihood (ML) reconstruction based on the selected model with a statistic of 5000 ultrafast bootstrap replications.

## Results

**Figure 2.**
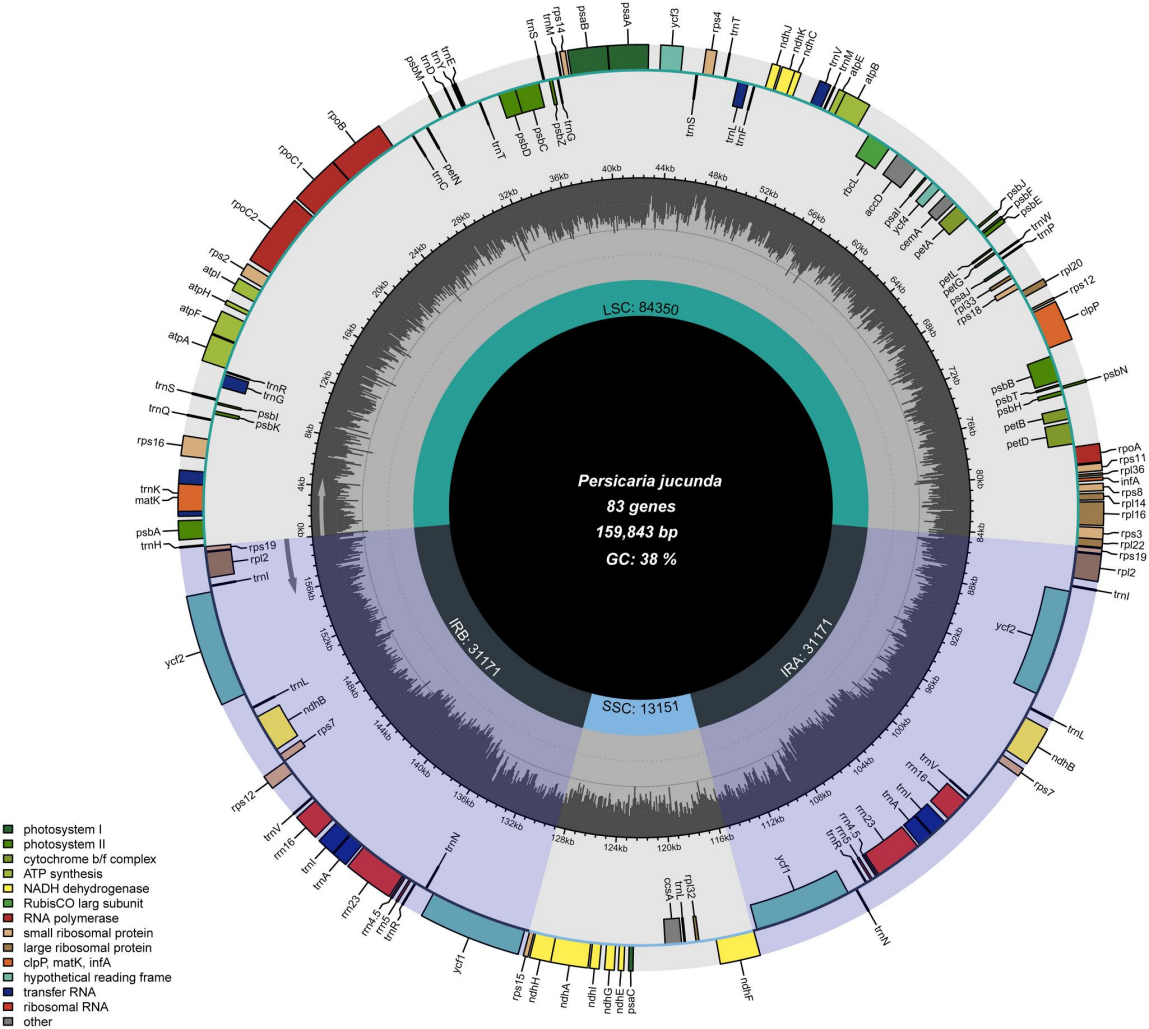
Circularized map of the *P. jucunda* chloroplast genome. Genes drawn outside the circle are transcribed counterclockwise, while those drawn inside the circle were clockwise. Color-code genes that belong to different functional groups.

Under the Linux operating system, the chloroplast genome of *P. jucunda* was assembled using GetOrganelle v1.7.7 (Jin et al. 2020) with default parameters. Coverage of the assembly results was calculated using a script provided by Cui et al. (2020) (Figure S1). The *P. jucunda* chloroplast genome is 159,795 bp in length, containing a large single-copy (LSC) region of 84,172 bp, a small single-copy (SSC) region of 13,420 bp, and two inverted repeat regions (IRs) of 31,102 bp (Figure 2). The total GC content is 37.9%, with the LSC region having a GC content of 36.1%, the SSC region having a GC content of 32.6%, and the IR region having a GC content of 32.6%. The total GC content was 37.9%, the LSC region GC content was 36.1%, the SSC region GC content was 36.1%, and the IR region GC content was 41.3%. The *P. jucunda* chloroplast genome contains 129 genes, including 85 protein-coding genes, 36 tRNA genes, and eight rRNA genes. The *rps12* is a trans-spliced gene (Figure A1). It has three unique exons. Two of them are duplicated as they are located in the IR regions. Eleven genes, including *rps16*, *atpF*, *ycf3*, *petB*, *petD*, *rpl16*, *rpl12*, *ndhB*, *ndhA*, *ndhB*, and *rpl2*, contain one or two introns (Figure A1). The pi value of nucleotide diversity ranged from 0 to 0.0158 (Figure A2). Seven regions with relatively high variability were determined as potential molecular markers or specific barcodes for species identification and phylogeny in the genus *Persicaria*. We found that the seven regions with relatively high nucleotide diversity, including *trnK*-*exon2*-*matK*, *trnE*-*trnT*, *psbC*-*trnS*, *rps4*-*trnT*, t*rnL*-*exon2*-*trnF*, *accD*-*psaI*, and *rpl32*-*trnL*, were all intergenic spacer regions.

**Figure 3.**
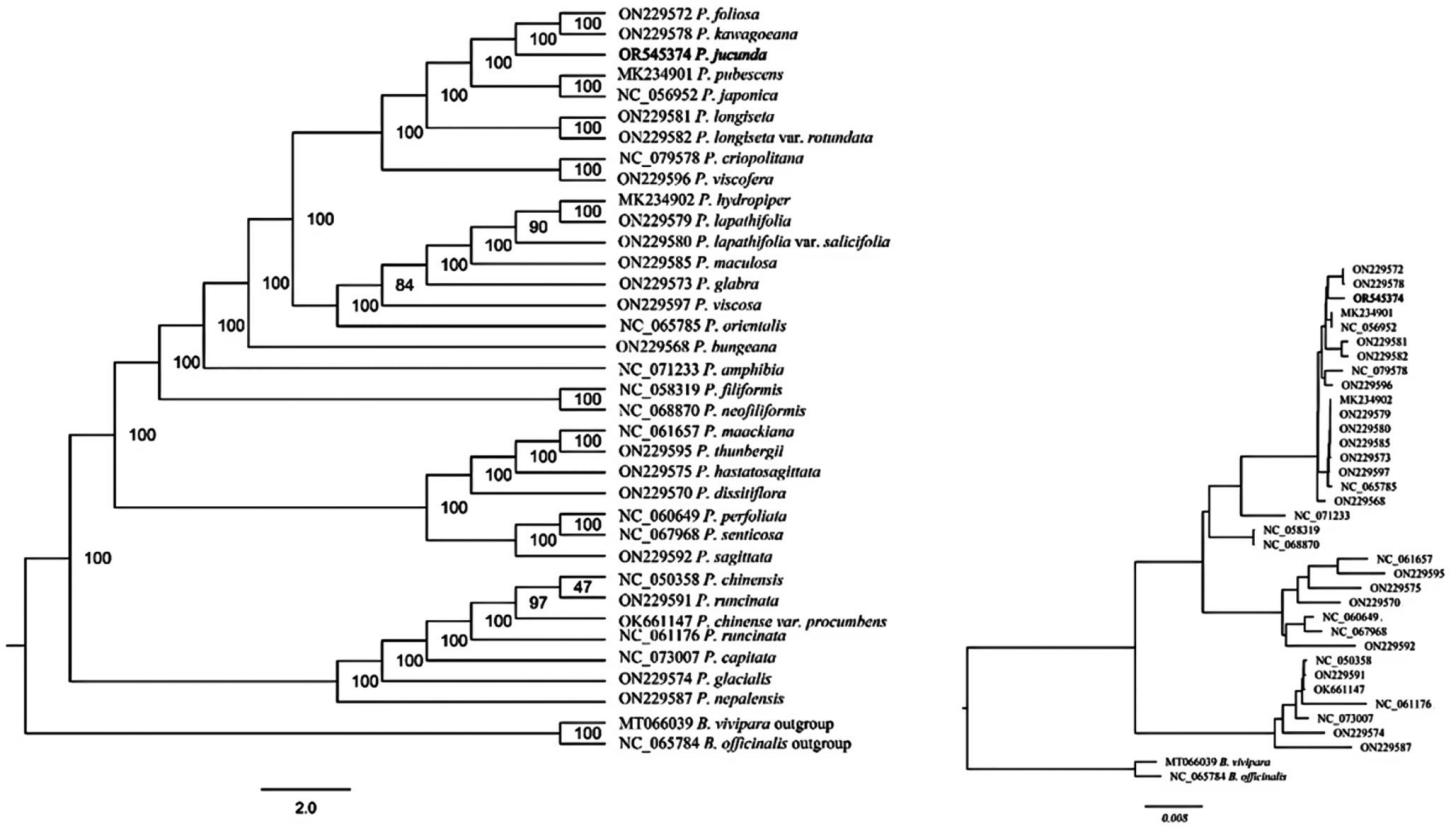
Phylogenetic position of *P. jucunda* inferred by maximum likelihood (ML) based on chloroplast genome sequence from 34 species from Polygonaceae with two species from Plumbaginaceae outgroup. Sequences used in this study were downloaded from the NCBI GenBank database. The bootstrap values are shown next to the nodes. The ‘sequences’ used were: *P. foliosa* ON229572 (Cao et al. 2022), *P. kawagoeana* ON229578 (Cao et al. 2022), *P. pubescens* MK234901, *P. japonica* NC_056952, *P. longiseta* ON229581 (Cao et al. 2022), *P. criopolitana* NC_079578, *P. viscofera* ON229596 (Cao et al. 2022), *P. hydropiper* MK234902 (Choi et al. 2022), *P. lapathifolia* ON229579 (Cao et al. 2022), *P. lapathifolia* var. *salicifolia* ON229580 (Cao et al. 2022), *P. maculosa* ON229585 (Cao et al. 2022), *P. glabra* ON229573 (Cao et al. 2022), *P. viscosa* ON229597 (Cao et al. 2022), *P. orientalis* NC_065785 (Guo et al. 2022), *P. bungeana* ON229568 (Cao et al. 2022), *P. amphibia* NC_071233 (Choi et al. 2022), *P. filiformis* NC_058319, *P. neofiliformis* NC_068870 (Zhang et al. 2022), *P. maackiana* NC_061657 (Kim et al. 2022), *P. thunbergia* ON229595 (Cao et al. 2022), *P. hastatosagittata* ON229575 (Cao et al. 2022), *P. dissitiflora* ON229570 (Cao et al. 2022), *P. perfoliata* NC_060649 (Yang et al. 2022), *P. senticosa* NC_067968, *P. sagittate* ON229592 (Cao et al. 2022), *P. chinensis* NC_050358 (Yu et al. 2020), *P. runcinate* ON229591 (Cao et al. 2022), *P. chinense* var. *procumbens* OK661147 (Zhang et al. 2022), *P. runcinate* NC_061176 (Choi et al. 2022), *P. capitata* NC_073007, *P. glacialis* ON229574 (Cao et al. 2022), *P. nepalensis* ON229587 (Cao et al. 2022), *B. vivipara* MT066039 (Marr et al. 2013), and *B. officinalis* NC_065784 (Guo et al. 2022).

To determine the phylogenetic positions of the newly described genomes, we downloaded 33 Polygonaceae chloroplast genomes and analyzed them phylogenetically using two of the Plumbaginaceae as outgroups (Figure 3). The results showed that the 34 genomes were divided into two major branches. *P. jucunda*, *P. foliosa*, and *P. kawagoeana*, were in the same branch, with *P. jucunda* and the other two being sister groups to each other. Within the genus, *P. jucunda*, *P. foliosa*, and *P. kawagoeana* are most closely related.

## Discussion and conclusions

The chloroplast genome, as a highly sequence-conserved hyper barcode, can provide information for resolving complex evolutionary relationships among species (Li et al. 2021; Yang et al. 2022). In this study, we report the complete chloroplast genome of *P. jucunda* for the first time. We found that the structural content of the organo chloroplast genome is highly conserved, with a tetrameric structure, protein-coding genes, tRNAs and rRNAs, and GC content similar to those of other angiosperms (Alzahrani et al. 2020; Du et al. 2020; Lee et al. 2023). This finding aligns with the general range of angiosperm chloroplast genomes (120–160 kb, encoding approximately 110–130 unique genes) (Dobrogojski et al. 2020), indicating that the genome size and gene content exhibit significant conservation. The application of chloroplast genome data in plant phylogenetic analysis is becoming increasingly widespread (Parks et al. 2009). Based on the existing chloroplast genome sequences, this study analyzed their phylogenetic relationships. The phylogenetic analysis indicates that *P. jucunda* forms a sister group with *P. foliosa*, *P. kawagoeana*, *P. pubescens*, and *P. japonica*, and is located at the most divergent end of the phylogenetic tree. These species are widely distributed in the region, suggesting that their ancestral group may have had a broad distribution in this area, potentially undergoing distinct evolution due to geographic isolation. The phylogenetic relationships of *P. jucunda* were analyzed by the complete chloroplast genome, providing valuable insights into the phylogeny and evolution of Polygonaceae.

## Supplementary Material

Supplemental Material

## Data Availability

The associated BioProject, SRA, GenBank ID, and Bio-Sample numbers are PRJNA1014382, SRR25951781, OR545374, and SAMN37323804, respectively.
