# Supplementary material for: The complete chloroplast genome and phylogenetic analysis of Persicaria jucunda (Meisn.) Migo (Polygonaceae)
Source: Mitochondrial DNA B Resour. 2025 Jan 28;10(2):144–8. doi: 10.1080/23802359.2025.2457450 (PMC11780699; doi:10.1080/23802359.2025.2457450)
Supplement: Supplemental Material [file TMDN_A_2457450_SM1762.docx]

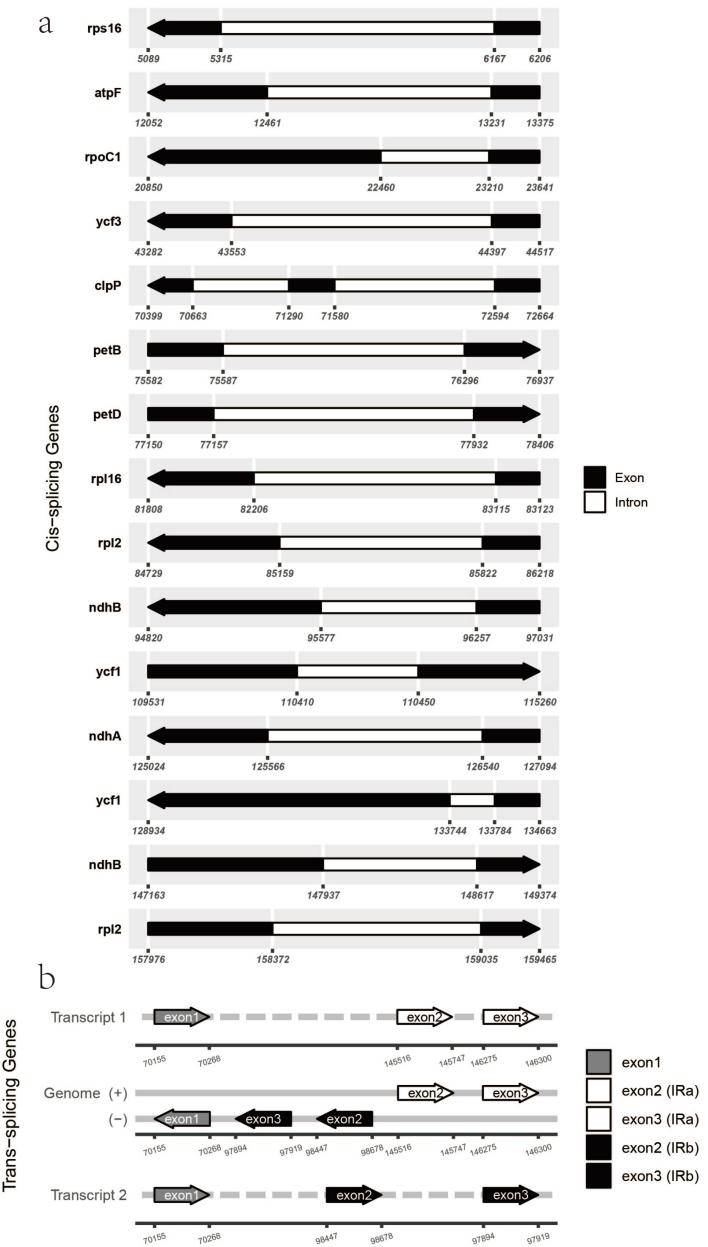


Fig Annex 1. Schematic map of the cis-splicing and trans-splicing genes. A, cis-splicing genes; B, trans-splicing genes. The arrow indicates the sense direction of the gene. Please note that lengths of exons and introns are not drawn to scale.


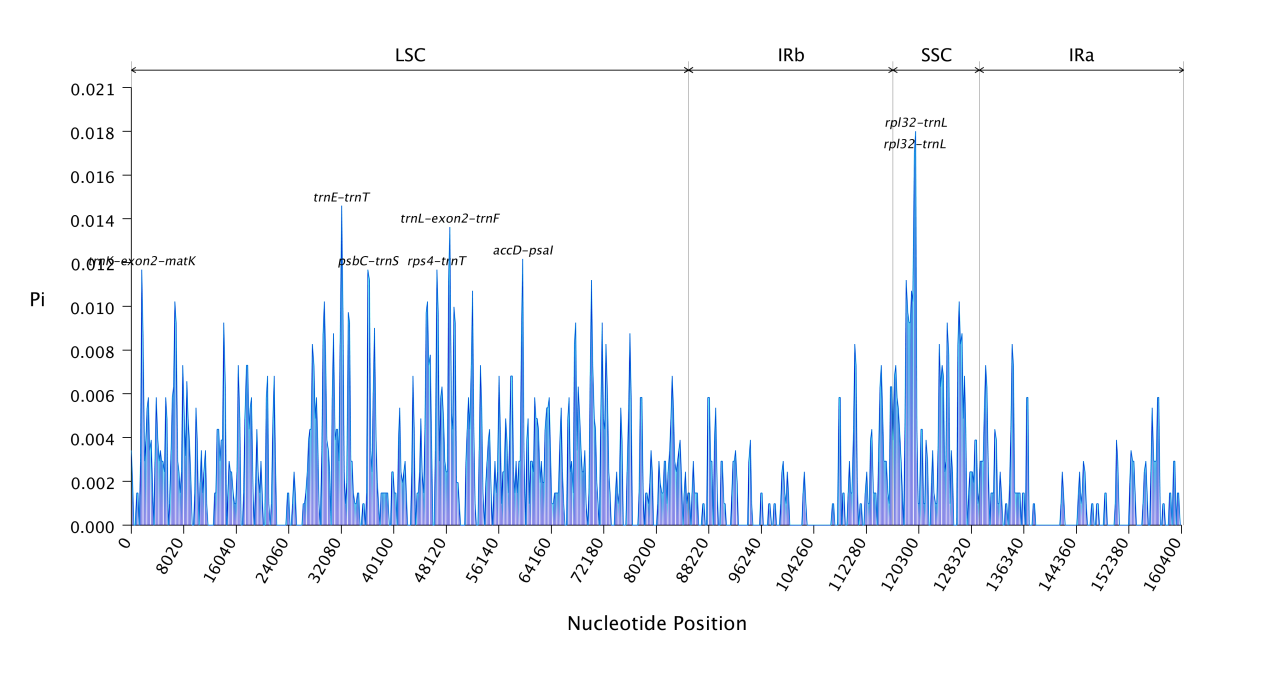


Fig Annex 2. Comparative analysis of the nucleotide polymorphism (Pi) values among the 5 cp genomes of Persicaria.


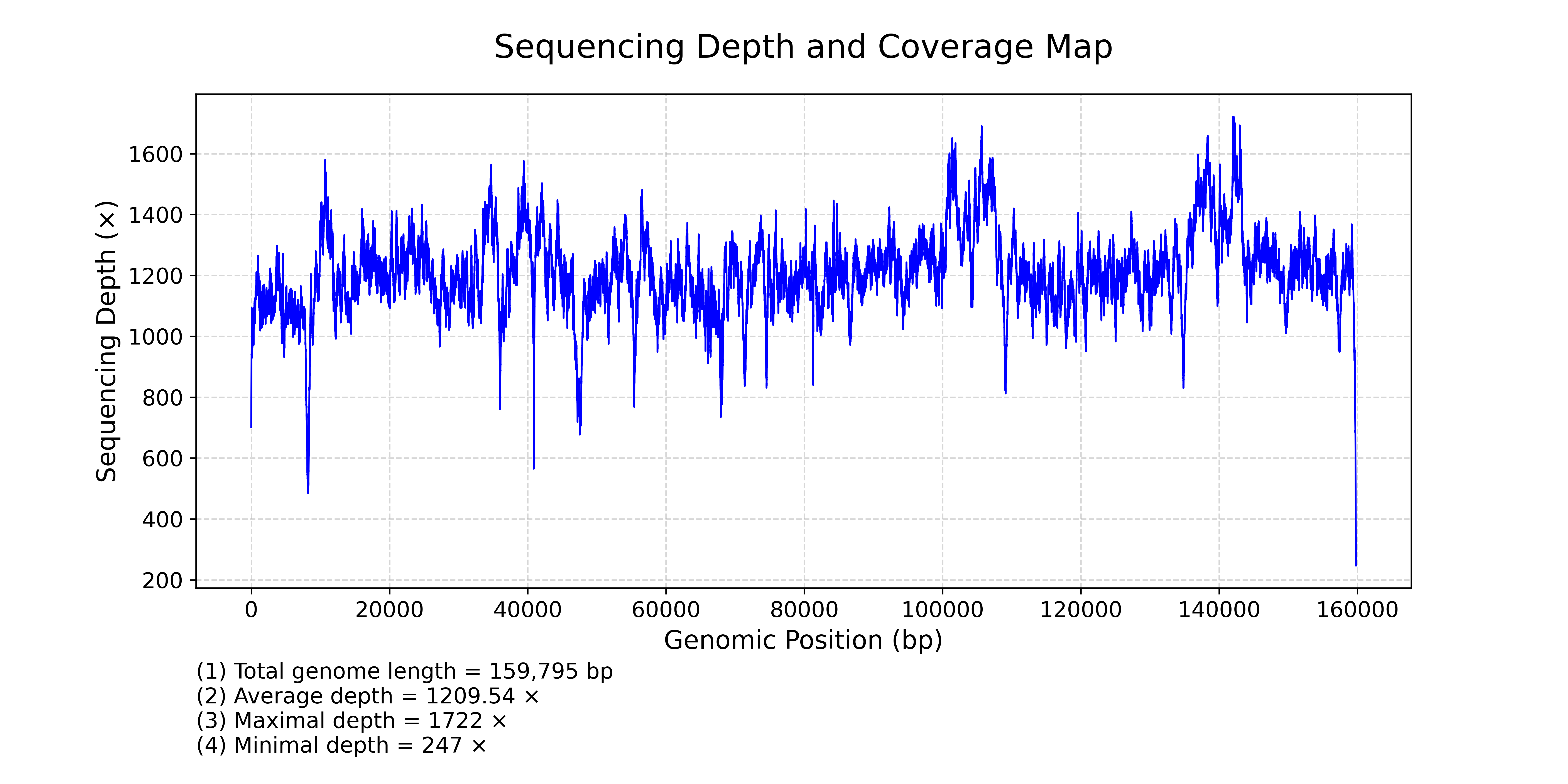


Fig S1. Sequencing Depth and Coverage Map. Coverage of the assembly results was calculated using a script provided by Ni et al (2023).
